# *Ilheus Virus* Isolate from a Human, Ecuador

**DOI:** 10.3201/eid1306.070118

**Published:** 2007-06

**Authors:** Barbara W. Johnson, Cristopher Cruz, Vidal Felices, William R. Espinoza, Stephen Robert Manock, Carolina Guevara, James G. Olson, Tadeusz J. Kochel

**Affiliations:** * Centers for Disease Control and Prevention, Fort Collins, Colorado, USA; † US Naval Medical Research Center Detachment, Lima, Peru; ‡ Hospital de la IV División del Ejercito “Amazonas,” Puyo, Ecuador; § Hospital Vozandes del Oriente, Shell, Ecuador

**Keywords:** Ilheus virus, Flavivirus, human infection, letter

**To the Editor:** *Ilheus virus* (ILHV) (genus *Flavivirus* in the Ntaya antigenic complex) is most closely related to *Rocio virus*. However, antibodies produced during ILHV infection cross-react in serologic assays to other flavivirus antigens, and ILHV was originally classified in the Japanese encephalitis antigenic complex (*1*–*3*). ILHV is transmitted in an enzootic cycle between birds and mosquitoes. Since the first isolation of ILHV from a pool of *Aedes* spp. and *Psorophora* spp. mosquitoes collected in 1944 at Ilheus City, on the eastern coast of Brazil (*4*), isolates have been obtained in Central and South America and Trinidad, primarily from *Psorophora ferox* mosquitoes (*5*,*6*). ILHV is not associated with epidemic disease and has been only sporadically isolated from humans (*5*,*7*–*9*). The clinical spectrum of human infections documented by virus isolation ranges from asymptomatic to signs of central nervous system involvement suggestive of encephalitis. Most commonly, patients exhibit a mild febrile illness accompanied by headache, myalgia, arthralgia, and photophobia, symptoms that may result in clinical diagnosis of dengue, Saint Louis encephalitis, yellow fever, or influenza (*7*). Laboratory diagnosis of ILHV infection may be difficult, unless a virus isolate can be obtained, because of the cross-reactivity in serologic assays to other flaviviruses that circulate in the same area, such as Rocio, dengue, yellow fever, and Saint Louis encephalitis viruses.

On March 1, 2004, after 4 days of symptoms, a 20-year-old male soldier stationed in Lorocachi, Ecuador, was admitted to the Hospital de la IV División del Ejercito “Amazonas” in Puyo, Ecuador. Lorocachi is in the Amazonian province of Pastaza, of which Puyo is the capital. The patient had fever, rash, epistaxis, headache, myalgia, retroocular pain, nausea, vomiting, jaundice, sore throat, and abdominal pain.

A blood specimen (containing isolate FSE800) was collected, and an acute-phase serum sample was processed for virus isolation in C6/36 (*Aedes albopictus*) and Vero cells. After 3 days, cytopathic effects were observed, and cells were screened by an immunofluorescence assay for reactivity against alphaviruses and flaviviruses by using polyclonal antibodies. The cells gave positive results with yellow fever, Saint Louis encephalitis, Rocio, and Ilheus antisera. Viral RNA was then extracted from the patient’s acute-phase serum and cell supernatants and processed for virus sequencing. Amplification of an ≈250-bp product by SyBRgreen real-time reverse transcriptase PCR and subsequent sequencing of the amplicon were conducted with the flavivirus consensus primers FU1 and cFD2 (*2*). Viruses were identified by BLAST search (www.ncbi.nlm.nih.gov/blast) and alignment to GenBank sequences. FSE800 had 96% identity (182 of 188 bp) with the nonstructural (NS) 5 region of the original ILHV strain AY632539 (*1*). An ILHV original 1944 isolate used as positive control had 100% sequence identity with AY632539.

Limited sequence information is available in GenBank for ILHV outside of the conserved NS5 region. Therefore, further sequencing of the NS5 region using flavivirus consensus primers FU1 and cFD3 (*2*) and the complete envelope (E) gene region, based on the complete open reading frame ILHV nucleotide sequence AY632539 (*1*), was conducted on FSE800, as well as a low-passage original 1944 isolate (R. Tesh, University of Texas Medical Branch [UTMB]), 2 mosquito isolates from Iquitos, Peru (R. Tesh, UTMB), and 2 human isolates from Brazil (CDC) (*8*) (Figure). There was 100% sequence identity among the 2 Brazilian and 2 Peruvian isolates at both gene regions. In the NS5 region, there was 100% identity between Brazilian and original isolates; 95% identity between FSE800 and Brazilian-original isolates; 98% identity between FSE800 and Peruvian isolates; and 96% identity between Peruvian and original-Brazilian isolates. At the protein level, 3 amino acid (aa) differences were found between FSE800 and original-Brazilian isolates, 2 between FSE800-Peruvian and original-Brazilian isolates (aa 3086, Lys-Gln; aa 3138, Ala-Val) and 1 between FSE800 and original-Brazilian-Peruvian isolates (aa 3309, Asp-Asn).

**Figure:**
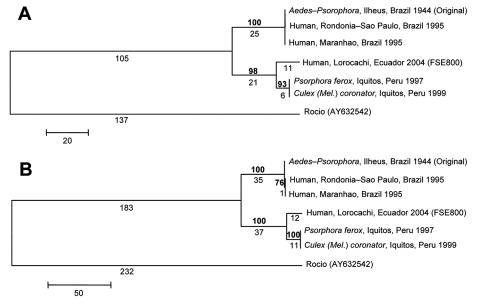
Phylogenetic analysis of the NS5 (A) and E gene (B) regions of 6 *Ilheus viru*s (ILHV) isolates. The sequences were aligned in MegAlign (DNASTAR, Inc., Madison, WI, USA); the alignments were then analyzed by using the maximum parsimony method with 500 bootstrap replicates in the program MEGA 3.1 (*10*). *Rocio virus* (GenBank accession no. AY632542) was included as an outgroup in the analysis, based on the phylogram of Kuno and Chang (*1*). Bootstrap values, shown in **boldface** above the branch, are percentages derived from 500 samplings; branch lengths are shown below the branch. The sequences generated from our study were deposited in GenBank under accession nos. EF396941–EF396952.

In the E gene region, there was 99.9% identity between original and Brazilian isolates; 95.1% and 95% identity between FSE800 and original and Brazilian isolates, respectively; 98.5% identity between FSE800 and Peruvian isolates; and 95.3% identity between Peruvian and original isolates. At the protein level, 2 aa differences were found between original-Brazilian and Peruvian-FSE800 isolates (aa 432, Ile-Thr; aa 652, Lys-Asn), 1 aa change only in Brazilian isolates (aa 570, His-Tyr), and 1 only in Peruvian isolates (aa 675, Asn-Ser). With the exception of the Ala-Val change, all were nonconservative changes. The effect of the amino acid changes has yet to be determined. However, nucleotide sequence comparison between the 6 ILHV isolates has shown that these gene regions are highly conserved geographically and temporally.

Ilheus virus is not usually associated with human disease, and human ILHV infections may not be correctly identified without a virus isolate because of the similar clinical symptoms and cross-reactivity in serologic assays to other flaviviruses. As laboratory surveillance is enhanced to monitor the emergence of West Nile virus in Central and South America, detection of ILHV and other flaviviruses may increase.
